# Factors Related to Blood Pressure Response after Community-Based Exercise Program in the Elderly Population

**DOI:** 10.3390/ijerph18063149

**Published:** 2021-03-18

**Authors:** Yi-Pin Wang, Kuo-Wei Tseng, Meng-Hui Lin, Mei-Wun Tsai

**Affiliations:** 1 Department of Physical Therapy and Assistive Technology, School of Biomedical Science and Engineering, National Yang Ming Chiao Tung University No.155, Sec.2, Linong Street, Beitou Dist., Taipei City 112304, Taiwan; ypw1202@gmail.com; 2 Department of Physical Therapy and Assistive Technology, National Yang Ming University No.155, Sec.2, Linong Street, Beitou Dist., Taipei City 112304, Taiwan; 3 Department of Exercise and Health Sciences, College of Kinesiology, University of Taipei No. 101, Sec. 2, Zhongcheng Rd., Shilin Dist., Taipei City 111036, Taiwan; fossil0405@yahoo.com.tw; 4 Department of Health, Taipei City Government No.1 City Hall Rd., Xinyi Dist., Taipei City 110204, Taiwan; mone@health.gov.tw; 5 Preventive Medicine Research Center, National Yang Ming Chiao Tung University No.155, Sec.2, Linong Street, Beitou Dist., Taipei City 112304, Taiwan

**Keywords:** blood pressure, health-related behavior, health promotion, physical activity, cardiovascular risks, community-based exercise program

## Abstract

Exercise has been recommended for blood pressure (BP) control, but not every individual can improve BP and reduce the risk of cardiovascular disease effectively by exercise. This study aimed to evaluate the BP response after 12-week exercise intervention and then identify the potential factors of responders on BP (R-BP) control. This was a retrospective cohort study from a project of Taipei City Government. Subjects completed the original program were included for further analysis. Sociodemographic factors, health-related behaviors, and cardiovascular risks were extracted as potential factors. The results were categorized into R-BP control, i.e., BP under optimal level (systolic BP (SBP) < 140 mmHg; and diastolic BP (DBP) < 90 mmHg) or a significant BP reduction (SBP ↓10 mmHg or DBP ↓5 mmHg) after intervention, or non-responder on BP control, i.e., subjects who failed to achieve the targets. There were 81.62% R-BP subjects. R-BP showed lower SBP and lower risk of hypertension at baseline. Active lifestyle could quadruple the number of R-BP. Higher educational level or more prescription medications were likely to be R-BP in subjects with diagnosed hypertension. Active lifestyle combined with exercise could benefit R-BP in the elderly population. Health-related factors also need to be considered for BP control.

## 1. Introduction

The aging population is growing fast in globalized countries, including Taiwan. Aging processes affect a broad range of tissues, organ systems, and functions, and the most important factor in aging is the increased risk of cardiovascular disease (CVD) [1]. The important CVD risk factors include hypertension (HTN), diabetes mellitus (DM), dyslipidemia, and obesity [2,3,4], of which HTN is the most prevalent and significant factor for CVD [5]. Previously, a meta-analysis study suggested that 54% and 47% cases of stroke and coronary artery disease, respectively, were attributed to HTN [6]. Recently, another meta-analysis study found that the relative risk of sudden cardiac death for people with and without HTN was 2.10. HTN and elevated systolic blood pressure (SBP) increased the risk of sudden cardiac death [7]. HTN is a modifiable factor. A study to assess the benefits of blood pressure (BP) lowering on recurrent stroke risk by geographic region found that treatment of HTN in patients with stroke led to a greater decrease in the recurrence of stroke in the Asian population compared with the Caucasian population. The absolute benefits were large in all treatment groups, particularly for the Asian population. Therefore, good control of HTN can effectively prevent the occurrence of CVD in Asians [8]. Preventive medicine suggested that maintaining at least <140 mmHg SBP and <90 mmHg diastolic BP (DBP) would be beneficial [9], and reducing SBP by 10 mmHg or DBP by 5 mmHg could decrease the risk of coronary artery disease and stroke by 22% and 41%, respectively [6].

Physical activity (PA) has always been critical to BP control. At least 150 min of moderate-intensity PA or 75 min of vigorous PA per week can effectively maintain health [10]. A systematic study proposed an association between PA and the risk of HTN. The risk of HTN was reduced by 7% with each 50 MET h/week (metabolic equivalent of task, MET) increment of total PA [11]. A longitudinal study has found that it is necessary to encourage people to engage in PA at relatively high level for effective BP control. This study used a questionnaire to obtain PA levels, and divided them into quartiles by MET. After adjustment for confounders, it was found that PA in the higher two quartiles corresponded to lower odds of HTN (12% and 15%, respectively; *p* < 0.05) compared with PA in the lowest quartile [12]. Exercise is a structured, purposeful type of PA [13] and is also recommended as the first-line nonpharmacological treatment of HTN in the prevention strategy [2,14]. The possible mechanism could be related to parasympathetic and sympathetic regulation and nitric oxide release, resulting in blood vessel dilation and insulin sensitivity that alter the relationship between vasodilatation- and vasoconstriction-related cytokines [15]. However, some individual differences were observed on BP control after exercise intervention [16]. Nearly 60% of 75 middle-aged subjects with a sedentary lifestyle, obesity, and prehypertension history had no change in BP control after six months of aerobic, resistance, or combined training [17]. A total of 182 elderly individuals with knee osteoarthritis and HTN were randomly allocated to the intervention group to receive an individual physical program or attention-control group to receive health education. The results showed no improvement in BP after the intervention [18]. A study of older women with HTN and normal BP found that almost half of the subjects experienced SBP reduction less than 2.58% after 10 weeks of resistance training [19]. Therefore, not everyone can effectively improve BP and reduce the risk of CVD after exercise.

The community is the base for healthcare service in urban and rural areas in Taiwan, and the community-based exercise program (CBEP) is generally acceptable, feasible for the elderly and can be popularized by people outside the healthcare system. Therefore, CBEP is an easier way to achieve health goals for all community-dwelling elderly [20]. CBEP included multiple components of exercise and health education that aimed to improve physical function, falling prevention, and health knowledge and benefited the social participation of elderly individuals [21,22]. This study aimed to evaluate the responsiveness of BP after CBEP regarding the primary prevention rule and to identify the possible underlying factors that cause individual differences on BP control after exercise intervention in the community setting. The first hypothesis was that CBEP would lead to significant improvement in BP in elderly individuals; the second hypothesis was that sociodemographic or health-related factors would be related to BP control.

## 2. Materials and Methods

### 2.1. Data Source and Study Design

This study was a secondary analysis with a retrospective cohort study design. The data were obtained from a pilot health promotion program of the Project of Taipei Advanced Center for Age and Health for elderly individuals. The University of Taipei Institutional Review Board approved the study (UT IRB: IRB-2020-045). In total, 172 participants were recruited from Song Shan District in 2016 through an advertisement posted in the local community and sports centers. All subjects lived in the community and were divided into five classes, according to their availability to join this project. The subjects’ inclusion criteria were as follows: age > 55 years, functional independence, and lack of cognitive impairment or dementia. Demographic data for all subjects were collected before 12 weeks CBEP, and cardiovascular risk markers were assessed prior to and after the intervention. The content of each class of the week was the same. In this secondary study, we extracted the data of subjects who were aged >65 years, had no history of CVD, and no missing data. Finally, 136 community-dwelling older adults were analyzed in this study.

### 2.2. Data Collection

The baseline assessment included sociodemographic factors, health condition, behavior, and cardiovascular risk markers. The sociodemographic variables included age, sex, body height, body weight, marital status (yes or no), educational level (below or above senior high school level), and living arrangement (alone or with family, friends, or others). The health condition and behavior-related variables involved self-reported disease (e.g., history of HTN, DM, hyperlipidemia, gastric and intestinal disease, cancer), number of prescription medications (0–1 per day, or >2 per day), routine health assessment (yes or no), smoking behavior (never, quit, or yes), drinking behavior (never or yes) and PA level. PA was measured using the International Physical Activity Questionnaire (IPAQ)-Taiwan version [23,24] by trained interviewers and divided into (1) inactive group and (2) sufficient-to-high activity group, according to the total energy consumption, as defined by the IPAQ. The cardiovascular risk markers consisted of resting BP, fasting plasma glucose (FPG) level, total cholesterol (TC) level, and body mass index (BMI). Resting BP was measured according to JNC 7 guidelines, which require the subjects in a sitting position after rest for 10 min with an automated sphygmomanometer [25]. FPG was measured after an overnight 10–12 h fasting. FPG and TC were measured with commercially available kits (Beckman Coulter, Brea, California, Beckman Coulter Inc., California, United States) [26]. BMI was calculated as weight/height^2^ (kg/m^2^). Weight was measured with participants standing without shoes and wearing light clothes to the nearest 0.1 kg on a calibrated scale. Height was also measured without shoes using a stadiometer to the nearest 0.1 cm. After the baseline assessment, a health center nurse explained the results of their health assessments and gave further advice and consultation.

### 2.3. Community-Based Exercise Program

Subjects received CBEP once a week for 12 weeks. The intervention programs were held in Song Shan health center and consisted of 10-min warm-up exercise, 40-min main exercise, and 10-min cool-down programs. The warm-up and cool-down programs included range of motion and stretching exercise of the spine and four extremities. The main training exercises consisted of endurance, balance, and strengthening exercises. Subjects performed stepping in place or up/down the stairs with rhythm as endurance exercise. Balance exercises included ball-playing in sitting and standing position or one-leg standing and tandem walking with or without hand support. Strengthening exercise was focused on core control and extremities using variable weight, resistance band, or body weight for resistance. To ensure consistency of the programs in each class, the intervention was introduced by one experienced physical therapist who demonstrated each exercise and ensured that the exercises were performed correctly. There was also an assistant during the exercise class to help the elderly follow the program. During the intervention period, no adverse events such as falls, sports injuries, or medical diseases occurred. All subjects signed in to record their attendance, and the adherence rate was calculated by dividing the actual attendance number by the total attendance number. In addition, in order to encourage the elderly individuals to participate, rewards were provided for attendance. Each of the 136 subjects participated more than 10 times, and the attendance rate exceeded 80%.

### 2.4. Definition of the Responder/Nonresponder on BP Control (R-BP/NR-BP)

The dependent variable was defined as the responsiveness of BP after the 12-week intervention, calculated using BP measured at baseline and final assessment. Based on the guideline of primary prevention and evidence of epidemiological study in the Seventh Joint National Committee, a decrease in CVD mortality and morbidity can be expected if individuals control their BP <140/90 mmHg and if there is a significant decrease of >10 mmHg in SBP or >5 mmHg in DBP [6]. Because of this reason, the subjects were categorized into R-BP if there was a decrease in BP from abnormal baseline data to normal range, decrease of >10 mmHg in SBP or >5 mmHg in DBP after the intervention, or maintenance of BP in the normal range. Otherwise, the subject was categorized as NR-BP.

### 2.5. Definition of Predictors

The independent variables were defined as predictors of BP control using sociodemographic data and health-related behaviors from the baseline assessment. The cardiovascular risk factors from the baseline assessment consisted of HTN risk, DM risk, hyperlipidemia risk and abnormal BMI risk. We defined HTN risk as baseline BP > 140/90 mmHg, according to the Seventh Joint National Committee hypertension guideline in 2003, DM risk as FPG level > 100 mg/dL, according to the American Diabetes Association in 2003, hyperlipidemia risk as TC level > 200 mg/dL, according to the National Cholesterol Education Program ATP III in 2001, and abnormal BMI risk as BMI < 18.5 kg/m^2^ or > 24 kg/m^2^, according to the Taiwan Ministry of Health and Welfare in 2002. If the subject showed increased BP, hyperglycemia, and hyperlipidemia simultaneously at baseline or had ever been diagnosed with DM or hyperlipidemia, it indicated that the subjects had clusters of abnormal cardiometabolic risk factors, which were classified as having triple H condition.

### 2.6. Statistical Analysis

SPSS 20.0 software (SPSS Inc., IBM, Chicago, IL, USA) was used for the statistical analysis. Normality of distribution for continuous variables was tested by the Kolmogorov–Smirnov test and Levene’s test of homogeneity of variances. Data were expressed as mean ± standard deviation (SD), median (mode), or count (percentage), as appropriate. Student’s paired *t*-test was used to identify the effect of health promotion programs on cardiometabolic risk factors. Independent t-test, Mann–Whitney U test, or chi square test was used to compare difference in predictors at baseline between R-BP and NR-BP. In addition, univariate logistic regression analysis was initially used to identify the potential predictors associated with R-BP if the *p*-value was ≤0.25. Afterward, the potential predictors were included in a multivariate logistic regression model to determine the independent factors of BP control after health promotion program. Nagelkerke’s R^2^ values were also specified. The statistical significance was set at a *p*-value <0.05.

## 3. Results

### 3.1. Characteristics of Subjects

There were 172 participants initially recruited from the community-based health promotion intervention project, but 36 subjects were excluded due to age < 65 years (*n* = 7), CVD history (*n* = 3), or incomplete data (*n* = 26). A total of 136 participants were finally analyzed in this study. There were 47 men (34.6%) and 89 women (65.4%), and the median (mode) age was 73 (67) years. Most elderly individuals were married and lived with family or friends. Moreover, 70.6% subjects had above senior high school educational level. In self-reported disease, HTN was the most common. A total of 44.1% subjects had HTN history and the others had no or unknown history. Furthermore, 52.2% subjects received 0–1 prescription medication per day. Few subjects did not receive regular health checks. A total of 90.4% subjects were classified in the sufficient-to-high activity group (Table 1). In CV risk at baseline, 78 (57.35%) subjects showed normal BP, 68 (50.00%) subjects showed normal TC level, and 62 (45.59%) subjects showed normal BMI, but only 36 (26.5%) subjects had normal FPG level. In addition, 21 (15.00%) subjects had clusters of cardiometabolic risks.

### 3.2. Comparison of the Potential Factors at Baseline of R-BP and NR-BP

**Figure 1:**
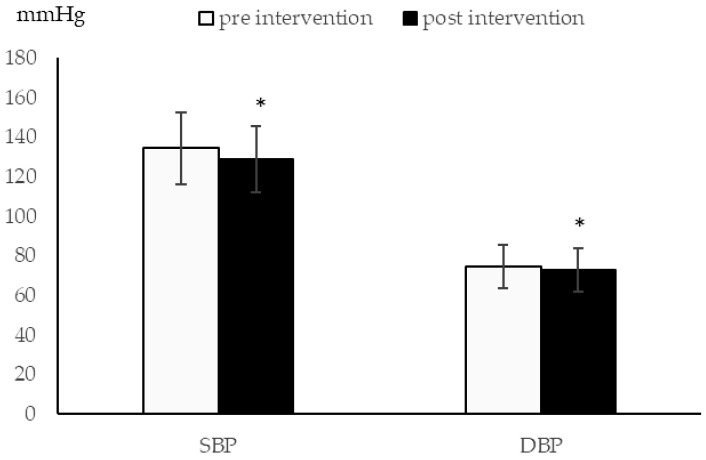
BP change after community-based exercise program. * Significantly different in intragroup comparison.

After the 12-week intervention, there was a significant improvement in SBP (before, 134.45 ± 18.25 vs. after, 128.72 ± 16.60, *p* < 0.01), DBP (74.50 ± 10.84 vs. 72.72 ± 10.92, *p* = 0.05) (Figure 1), TC level (207.07 ± 54.72 vs. 184.89 ± 34.52, *p* < 0.01), and BMI (23.90 ± 3.57 vs. 23.69 ± 3.49, *p* = 0.03), but not in FPG level (121.79 ± 35.46 vs. 120.72 ± 46.82, *p* = 0.75). Regarding the performance of BP control, 111 subjects (81.62%) and 25 subjects (18.38%) were classified as R-BP and NR-BP, respectively. There was no significant difference in sociodemographic and health-related factors between R-BP and NR-BP. However, the R-BP group had significantly lower SBP and lower risk of HTN and triple H condition (Table 2).

### 3.3. Factors Affecting R-BP

In univariate logistic regression, we found that baseline PA level, HTN risk, and cardiometabolic risk were factors related to R-BP and NR-BP. After adjusting for sex, educational level, PA level, HTN risk, and cardiometabolic risk, the active group was significantly more likely to be R-BP than the inactive group (odds ratio (OR) = 4.50; 95% confidence interval (CI): 1.19–17.02). Participants with HTN risk at baseline were less likely to be R-BP (OR = 0.37; 95% CI, 0.13–1.11), although this did not reach a significant level after adjusting for sociodemographic and health-related factors (Table 3).

### 3.4. Impact of HTN History on Factors Affecting R-BP

We further stratified subjects according to history of HTN. There were 60 participants diagnosed with HTN and 76 participants had no or unknown history. In the diagnosed group, 12 (20%) were classified as NR-BP after intervention and 48 (80%) were R-BP. In the non-diagnosed group, 13 (17.10%) were NR-BP, and 63 (82.89%) were R-BP. There was no significant difference between the R-BP and NR-BP groups in sociodemographic and health-related factors regarding diagnosed or non-diagnosed HTN, although educational level in the HTN-diagnosed group reached a significant level. In subjects with non-diagnosed HTN, the responder group showed significantly lower DBP, and the proportion of combined triple H condition was borderline significant (*p* = 0.06) regarding CV risk (Table 2).

In the univariate logistic regression analysis of subjects with a diagnosis of HTN, we found that educational level was a factor related to R-BP and NR-BP. After adjusting for sex, educational level, medication, and HTN risk at baseline, this factor still reached a significant level. Participants with higher educational level were significantly more likely to be R-BP than those with lower educational level (OR = 5.36; 95% CI, 1.05–27.34). After adjusting for living status, medication, and PA level in subjects with non-diagnosed HTN, participants with combined triple H condition were less likely to be R-BP than those without triple H condition (OR = 0.36; 95% CI, 0.08–1.59). In addition, it was interesting to note that the number of prescription medications used showed contradictory findings in these two groups. Subjects who received more than two drugs daily were likely to be R-BP than those who received less than two drugs daily in the HTN diagnosed group (OR = 2.37; 95% CI, 0.51–11.08). Nevertheless, it seemed to be a negative factor for R-BP in subjects with non-diagnosed HTN (OR = 0.45; 95% CI: 0.12–1.64) (Table 3).

## 4. Discussion

The 12-week community-based health promotion program could be effective for BP reduction, and elderly individuals with active lifestyle were more likely to be an R-BP. Furthermore, some disparities were found in subjects with or without HTN. In subjects with HTN, those with higher educational level had good response on BP control. In subjects with non-diagnosed HTN, those with clusters of abnormal cardiometabolic risks had less response to BP.

The effectiveness of exercise training in lowering BP is well confirmed [27]. A review study revealed that the PA programs that persisted for 2–7 sessions per week and had a total time of 90–165 min each week for 12 weeks to 3 years could lower BP in independent elderly individuals [28]. Another systematic review with meta-analysis on adults showed that an intervention duration of ≥24 weeks with resistance exercise training reduced SBP (−5.08 mm Hg) and DBP (−4.93 mm Hg); less change in BP was observed when the intervention duration was of <24 weeks [29]. Some individuals did not show responsiveness to BP after exercise in the current study. CBEP was relatively gentle; therefore, it was feasible and accessible for the elderly population [30]. The attendance rate of this study was >80%, which implies that participants had high acceptance of the program. In this study, CBEP was held for 60 min per week and once a week for 12 weeks. This implied that the exercise dose was inadequate to control BP well in some individuals. Thus, the main effect of CBEP was maintaining or improving physical function, health awareness, and social participation for elderly individuals, which is not obviously shown in individualized responsiveness to BP control. Regularly engaging in moderate to vigorous PA has a major role in BP control. Higher levels of PA leaded to lower cardiovascular events after long term follow up. In that study, the PA measurement was conducted by PA questionnaire which was a single question with four levels only (inactivity, light activity, moderate activity, and high-level activity) to measure leisure-time PA. Therefore, it might not able to measure total PA comprehensively. [31]. However, currently, lifestyle modification is considered the most important factor in controlling BP because of safety, low cost, and effectiveness in prevention and management of HTN [32,33]. A large-scale prospective cohort study from the United Kingdom supports the role of lifestyle factors to eliminate undesirable BP genetic factors and reduce the risk of CVD [34].

Our findings revealed that SBP and DBP decreased by ~5.73 mmHg and 1.78 mmHg, respectively. The reduction was lower than in previous studies. One study found a significant improvement in SBP and DBP (−8/−5.5 mmHg) after high-intensity interval training for 16 weeks in prehypertensive women in Chile who were overweight or obese with sedentary lifestyle, but this difference was not observed in normal tension group [35]. A meta-analysis study also showed that the net change in SBP and DBP after aerobic exercise was greater in hypertensive subjects (−8.3/−5.2 mmHg) than in prehypertensive (−2.1/−1.7 mmHg) and normotensive subjects (−0.75/−1.1 mmHg) [36]. Therefore, the result of BP decrease could possibly be explained from primary BP health status. In addition, decreased SBP would be related to the baseline value of SBP [37]. A randomized controlled trial of a 6-month lifestyle intervention group vs. a control group in southern California found that BP at baseline was significantly associated with BP change after intervention in community-dwelling elderly with HTN [38]. However, we did not find an association between prior BP health and BP change after adjustment. BP reduction in this study was smaller and may be attributed to the classification of participants in the prehypertensive stage at the beginning; however, BP improved to normal after CBEP in this study.

Comorbidity may lead to poor health-related outcomes [39]. Subjects with metabolic syndrome may contribute to lower BP reduction after PA treatment [40,41]. The change in BP during exercise is strongly associated with serum TC level and insulin resistance [42]. Therefore, poor control of HTN, hyperglycemia, and hyperlipidemia may lead to less response to CV risk control after the intervention.

Favorable changes in education accounted for improved control of HTN and its prevention [43]. In our study, higher educational level was a major factor of good BP control in subjects with HTN. In the USA, individuals with education above senior high school educational level have lower prevalence of HTN. Those with higher education usually have better health awareness and regularly receive health assessment. They are also more likely to provide an accurate self-report of HTN [44]. Moreover, a contrasting finding was observed. The number of prescription medications in the responder group demonstrated a reverse tendency in subjects with diagnosed and non-diagnosed HTN, although this situation did not reach statistical significance. Due to inadequate information about the medical effects in the questionnaire, we speculated that subjects with HTN may have received antihypertensive medication. In subjects with non-diagnosed HTN, the number of prescription medications may indicate that other complications may have existed that required medical intervention but not BP control. This finding indirectly proved the efficacy of the medication in these two groups. However, due to insufficient sample size and lack of related records, the inference is worthy of confirmation in the future.

This study had some limitations. First, the sample size and characteristics of the participants were limited. A minimum of 182 participants were required with a power of 0.80 after calculation by using G*power 3.1.9.7 (Heinrich-Heine-Universität Düsseldorf, Düsseldorf, Germany). Hence, more subjects from more communities should be included in future studies to verify the results of this study. In addition, the characteristics of enrolled participants regarding active lifestyle with better motivation limit the generalizability to other populations. Second, participants with CVD history were excluded so the outcome may be implicated for primary prevention and did not target various clinical conditions, such as history of diabetes and other metabolic diseases. Third, BP was measured at specific times rather than consecutively for an extended period, and the interpretation of BP control may be over- or underestimated. Fourth, insufficient information from the original data source limited further exploration. Dietary record and detailed information regarding medication use may need to be added during the intervention period to distinguish the real role in BP control. In addition, the original project did not require the subjects to not engage in other activities, and IPAQ was only collected at baseline; therefore, there is a lack of records of PA outside the intervention period and exercise intensity records during the intervention. Sensor-based PA monitoring for evaluating the dose-response effects and rating of perceived exertion records should be provided during the intervention in future studies. In this study, the intervention period was relatively short. Therefore, whether it can be applied to other studies with longer-term intervention should be carefully considered.

## 5. Conclusions

Active lifestyle combined with CBEP could lower BP to effectively reduce the risk of cardiovascular diseases. Community-dwelling elderly should be encouraged to participate in more PA to enhance the effect of BP control after CBEP. When evaluating the effect of BP control of health promotion policy for the community-dwelling elderly, PA level, educational level, and prescription medications of the participants should also be taken into consideration.

## Figures and Tables

**Table 1:** Sociodemographic and health-related information at baseline.

*n* = 136	Number	Percent (%) Mean ± SD/Median (Mode)
Sociodemographic Information		
Sex	Male 47/Female 89	34.6/65.4
Age (year)		73 (67)
Body height (cm)		155.80 (155)
Body weight (kg)		57.80 (52.20)
BMI		23.90 ± 3.56
Marital status		
Married	92	67.6
Widow/single/divorce	44	32.4
Living arrangements		
Living with family/friends	110	80.9
Living alone	24	17.6
Others	2	1.5
Highest level of education		
Less than senior high school level	40	29.4
Senior high school level	46	33.8
College or advance degree	50	36.8
Health condition- and behavior-related Information		
Self-reported disease		
HTN	60	44.1
GI illness	17	12.5
DM	17	12.5
Immune disease	8	5.9
Hyperlipidemia	5	3.7
Pulmonary disease	4	2.9
Prostate hypertrophy	4	2.9
Thyroid disease	3	2.2
Renal disease	2	1.5
Liver disease	1	0.7
Cancer	1	0.7
Not reported	14	10.4
Prescription medications		
0–1	71	52.2
2–3	47	34.6
≥4	18	13.2
Health assessment in the past year		
No	21	15.4
PA level		
Inactive lifestyle	13	9.6
Active lifestyle	123	90.4
Smoking		
Never	123	90.4
Quit	11	8.1
Yes	2	1.5
Drinking		
Never	99	72.8
Yes	37	27.2

HTN, hypertension; DM, diabetes mellitus; GI, gastrointestinal; PA, physical activity.

**Table 2:** Comparison of the potential factors at baseline of responder/non-responder in all subjects and subjects with or non-diagnosed HTN.

		All Subjects (*n* = 136)		With HTN (*n* = 60)		Non-Diagnosed HTN (*n* = 76)	
Baseline Data		NR-BP (*n* = 25) Number (%)	R-BP (*n* = 111) Number (%)	NR-BP (*n* = 12) Number (%)	R-BP (*n* = 48) Number (%)	NR-BP (*n* = 13) Number (%)	R-BP (*n* = 63) Number (%)
Sociodemographic factors							
Age ^a^		74(72)	73(67)	77(77)	73.5(73)	73(69)	72(67)
Sex	Male	6(24)	41(36.9)	2(16.7)	18(37.5)	4(30.8)	23(36.5)
	Female	19(76)	70(63.1)	10(83.3)	30(62.5)	9(69.2)	40(63.5)
Educational level	Below senior high school level	10(40)	30(27)	8(66.7)	14(29.2)	2(15.4)	16(25.4)
	Above senior high school level	15(60)	81(73)	4(33.3)	34(70.8) *	11(84.6)	47(74.6)
Living arrangements	Living alone	5(20)	19(17.1)	2(16.7)	12(25)	3(23.1)	7(11.1)
	Living with family/friends	20(80)	90(81.1)	10(83.3)	36(75)	10(76.9)	54(85.7)
	Others	0(0)	2(1.8)	0(0)	0(0)	0(0)	2(3.2)
Health condition and behavior-related factors							
Medication	0–1	12(48)	59(53.2)	5(41.7)	11(22.9)	7(53.8)	48(76.2)
	≥2	13(52)	52(46.8)	7(58.3)	37(77.1)	6(46.2)	15(23.8)
Health examination	No	4(16)	17(15.3)	2(16.7)	8(16.7)	2(15.4)	9(14.3)
	Yes	21(84)	94(84.7)	10(83.3)	40(83.3)	11(84.6)	54(85.7)
PA level	Inactive lifestyle	5(20)	8(7.2)	2(16.7)	4(8.3)	3(23.1)	4(6.3)
	Active lifestyle	20(80)	103(92.8)	10(83.3)	44(91.7)	10(76.9)	59(93.7)
Cardiovascular risk factors							
BP at baseline	SBP (mmHg)	141.16 ± 16.37	132.94 ± 18.38*	145.16 ± 16.73	139.08 ± 19.44	137.46 ± 15.765	128.26 ± 16.17
	DBP (mmHg)	76.80 ± 10.18	73.99 ± 10.96	76.25 ± 12.15	77.87 ± 10.22	77.30 ± 8.44	71.0 ± 10.65*
	No risk	9(36)	69(62.2)	3(25)	24(50)	6(36.2)	45(71.4)
	Elevated in BP	16(64)	42(37.8) *	9(75)	24(50)	7(53.8)	18(28.6)
FPG level at baseline	FPG (mg/dL)	125.16 ± 40.54	121.03 ± 34.3	127.08 ± 49.00	120.60 ± 29.40	123.38 ± 32.84	121.36 ± 37.96
	No risk	5(20)	31(27.9)	2(16.7)	11(22.9)	3(23.1)	20(31.7)
	Impaired	12(48)	47(42.3)	6(50)	24(50)	6(46.2)	23 (36.5)
	DM	8(32)	33(29.7)	4(33.3)	13(27.1)	4(30.8)	20(31.7)
TC level at baseline	TC (mg/dL)	202.76 ± 43.26	208.04 ± 57.10	189.33 ± 42.91	192.83 ± 47.76	215.15 ± 41.34	219.63 ± 61.16
	No risk	11(44)	57(51.4)	7(58.3)	27(56.2)	4(30.8)	30(47.6)
	Borderline	9(36)	29(26.1)	3(25)	13 (27.1)	6(46.2)	16(25.4)
	Hyperlipidemia	5(20)	25(22.5)	2(16.7)	8 (16.7)	3(23.1)	17(27.0)
BMI at baseline	BMI	24.56 ± 3.38	23.76 ± 3.60	25.64 ± 3.79	24.98 ± 3.39	23.56 ± 2.72	22.82 ± 3.50
	No risk	11(44.0)	51(45.9)	5(41.7)	17(37.4)	6(46.2)	34(54.0)
	Under/overweight	7(28.0)	38(34.2)	3(25)	17(35.4)	4(30.8)	21(33.3)
	Obesity	7(28.0)	22(19.8)	4(33.3)	14(29.2)	3(23.1)	8(12.7)
Triple H	No triple H	18(72)	97(87.4)	9(75)	41(85.4)	9(69.2)	56(88.9)
	Combine triple H	7(28)	14(12.6) *	3(25)	7(14.6)	4(30.8)	7(11.1) *p* = 0.06

HTN, hypertension; BP, blood pressure; R-BP, responder of BP control; NR-BP, non-responder on BP control; PA, physical activity; FPG, fasting plasma glucose; TC, total cholesterol. * Significantly different in intergroup comparison. ^a^ Data was expressed as median (mode).

**Table 3:** Odds ratio (OR) and 95% confidence interval (CI) of independent predictors in R-BP by logistic regression.

	All Subjects (*n* = 136)		With HTN (*n* = 60)		Non-Diagnosed HTN (*n* = 76)	
Predictor at Baseline	Univariate	Multivariate	Univariate	Multivariate	Univariate	Multivariate
	OR (95%CI)	OR (95%CI)	OR (95%CI)	OR (95%CI)	OR (95%CI)	OR (95%CI)
Sociodemographic factors						
Age	0.96		0.94		0.99	
	(0.90–1.03)		(0.85–1.05)		(0.89–1.09)	
Sex (Female)	0.54	0.52	0.33	0.94	0.77	
	(0.20–1.46)	(0.17–1.53)	(0.07–1.70)	(0.13–6.72)	(0.21–2.79)	
Educational level (Above senior high school)	1.80	1.65	4.86	5.36	0.53	
	(0.73–4.44)	(0.61–4.47)	(1.26–18.77)	(1.05–27.34)	(0.11–2.67)	
Living arrangements (Living with company)	1.21		0.60		2.40	1.62
	(0.40–3.63)		(0.12–3.13)		(0.53–10.87)	(0.31–8.57)
Health condition and behavior-related factors						
Medications (≥2)	0.81		2.40	2.37	0.37	0.45
	(0.34–1.94)		(0.64–9.09)	(0.51–11.08)	(0.11–1.25)	(0.12–1.64)
Health examination (Yes)	1.05		1.00		1.09	
	(0.32–3.45)		(0.18–5.46)		(0.21–5.76)	
PA level	3.22	4.50	2.20		4.43	3.07
(Active lifestyle)	(0.95–10.86)	(1.19–17.02)	(0.35–13.73)		(0.86–22.81)	(0.50–18.67)
Cardiovascular risk factors						
HTN risk	0.34	0.37	0.33	0.31	0.34	
	(0.14–0.84)	(0.13–1.11)	(0.08–1.38)	(0.07–1.44)	(0.10–1.16)	
DM risk	0.65		0.67		0.65	
	(0.22–1.87)		(0.13–3.54)		(0.16–2.60)	
Hyperlipidemia risk	0.74		1.09		0.49	
	(0.31–1.78)		(0.30–3.92)		(0.14–1.75)	
Abnormal BMI risk	0.92		1.30		0.73	
	(0.39–2.21)		(0.36–4.73)		(0.22–2.42)	
Cardiometabolic risk (combined triple H)	0.37	0.62	0.51		0.28	0.36
	(0.13–1.05)	(0.19–2.06)	(0.11–2.37)		(0.07–1.16)	(0.08–1.59)
Nagelkerke’s R2		0.15		0.24		0.15

OR, odds ratio; CI, confidence interval; R-BP, responder of BP control; HTN, hypertension, PA, physical activity; CV, cardiovascular; DM, diabetes mellitus.

## Data Availability

The authors confirm that the data supporting the findings of this study are available within the article.
